# Synthetic Processes toward Nitriles without the Use of Cyanide: A Biocatalytic Concept Based on Dehydration of Aldoximes in Water

**DOI:** 10.1002/chem.202001647

**Published:** 2021-01-22

**Authors:** Alessa Hinzmann, Tobias Betke, Yasuhisa Asano, Harald Gröger

**Affiliations:** ^1^ Chair of Industrial Organic Chemistry and Biotechnology Faculty of Chemistry Bielefeld University Universitätsstraße 25 33615 Bielefeld Germany; ^2^ Biotechnology Research Center Toyama Prefectural University 5180 Kurokawa Imizu Toyama 939-0398 Japan

**Keywords:** bioorganic chemistry, enantioselectivity, enzyme catalysis, nitriles, synthesis design

## Abstract

While belonging to the most fundamental functional groups, nitriles represent a class of compound that still raises challenges in terms of an efficient, cost‐effective, general and, at the same time, sustainable way for their synthesis. Complementing existing chemical routes, recently a cyanide‐free enzymatic process technology based on the use of an aldoxime dehydratase (Oxd) as a biocatalyst component has been developed and successfully applied for the synthesis of a range of nitrile products. In these biotransformations, the Oxd enzymes catalyze the dehydration of aldoximes as readily available substrates to the nitrile products. Herein, these developments with such enzymes are summarized, with a strong focus on synthetic applications. It is demonstrated that this biocatalytic technology has the potential to “cross the bridge” between the production of fine chemicals and pharmaceuticals, on one hand, and bulk and commodity chemicals, on the other.

## Graphical abstract

**Nitrile synthesis with enzymes**: An overview of a biocatalytic process technology suitable for the efficient and cyanide‐free synthesis of nitriles by means of dehydration of aldoximes in water is given. A broad range of nitriles are obtained in such biotransformations. Chiral nitriles can be obtained in high enantiomeric excess. The aldoxime substrates are readily available by condensation of aldehydes with NH_2_OH.

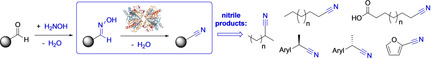

## Introduction

Rapid depletion of our global resources, which is also true for noble metals, prompts us to rethink the production methods for many of today's chemical compounds. Increasing product demand in all segments of the chemical industry forces us to develop reliable and, at the same time, sustainable production processes, which can meet our needs now and in the future. Biocatalysis is considered to represent one of the key technologies for enabling such processes.[^1^, ^2^, ^3^, ^4^, ^5^, ^6^] Often biocatalytic processes run under milder conditions, compared with many chemical processes, and excel at selectivity, and the access to biocatalysts is not affected by limited raw‐material sources, as in case of a range of precious metals used in chemocatalysis. Whereas the economy of such metal‐catalyzed processes often depends on varying price and availability of the corresponding metal, biocatalysts can be simply produced by fermentation. Additionally, precious metals need to be efficiently recycled and have to be restricted in their exposure towards animals, humans, and environment due to their, at least in part, high toxicity. Biocatalysts, on the other hand, are completely biodegradable and can be easily produced under optimized cultivation procedures. However, the successive implementation of biocatalytic processes into the chemical industry should always be regarded and used as an alternative to complement chemocatalytic processes.^[7]^ Thus, biocatalysis, as such an additional alternative, should be viewed as an opportunity for broadening the chemical repertoire and not as the all‐promising solution to every synthetic problem. In abiding by these standpoints, new and fascinating possibilities open up.

Today, mainly four approaches are used in the chemical industry for nitrile synthesis, depending on the structure and application of the produced nitrile.[^8^, ^9^] One of the most important production processes for nitriles by annual tonnage is the double hydrocyanation of 1,3‐butadiene to yield adiponitrile. Adiponitrile is a key intermediate in nylon production and is almost exclusively hydrogenated to hexamethylenediamine. In addition, adiponitrile is synthesized by electro‐hydrodimerization of acrylonitrile. Acrylonitrile, an unsaturated nitrile, is accessible by means of another important industrial process technology for nitrile synthesis: ammoxidation. This process utilizes unsaturated hydrocarbons, ammonia, and air to yield the corresponding nitrile. Because ammoxidation benefits from the easy modification of C−H bonds next to a C=C‐moiety, aromatic nitriles (starting from toluene derivatives) are also accessible. Lastly, amides can be dehydrated towards their corresponding nitriles under elevated temperatures and in the presence of heterogeneous catalysts. This process is mostly used for the synthesis of fatty nitriles due to the high accessibility of long‐chain, aliphatic fatty acids as starting materials. The formed fatty nitriles are hydrogenated (mainly heterogeneously) towards fatty amines, which are used as surfactants or lubricant additives. If one evaluates these processes, in spite of their impressive utilization on a large scale, several drawbacks are also apparent. For example, highly toxic hydrogen cyanide has to be used for hydrocyanation approaches. Moreover, fatty amide formation and dehydration to fatty nitriles from fatty acids represents a tedious and energy‐intensive process with excessive temperatures of around 300 °C to proceed efficiently. Comparable harsh conditions are also applied in the gas‐phase ammoxidation process, which raises selectivity and side‐product formation concerns. As for ammoxidation, acetonitrile and hydrogen cyanide can be formed as side products, whereas the double hydrocyanation of butadiene also leads to regioisomers of adiponitrile. In the case of amide dehydration, purification by distillation is of paramount importance to obtain the nitrile in high purity.

**Scheme 1:**
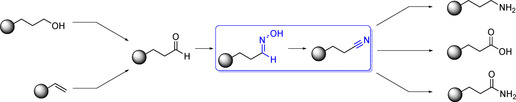
Alternative synthetic route towards nitriles by using aldehydes accessed by hydroformylation of alkenes or oxidation of alcohols as starting materials. Aldehydes are condensed with hydroxylamine to give aldoximes, which can subsequently serve as substrates for Oxd‐catalyzed nitrile synthesis. Nitriles can later be used for several transformations, resulting in valuable chemical products.

Avoiding the abovementioned drawbacks, a biocatalytic concept based on the dehydration of aldoximes in water, thus yielding the desired nitriles with high selectivity, has recently turned out to be a promising approach for a mild, sustainable nitrile synthesis. The required aldoximes are easily accessible by condensation of the bulk chemical hydroxylamine with aldehydes (Scheme 1). The required aldehydes are themselves broadly accessible by homogeneous catalyzed hydroformylation of alkenes with syngas or by oxidation of alcohols by means of, for example, a (piperidin‐1‐yl)oxyl radical as a catalyst and sodium hypochlorite as an oxidation agent.[^10^, ^11^] The enzymes utilized for this biotransformation are the aldoxime dehydratases (Oxds). Oxds, belonging to the enzyme class of lyases (EC 4.99.1.5–4.99.1.7), were first described in 1998 by Asano et al., and their high potential for organic synthesis has just recently been intensively explored.[^12^, ^13^, ^14^, ^15^, ^16^, ^17^, ^18^, ^19^] The independence of such enzymes from the need for cofactors and already high specific activity of the wild‐type enzymes for many substrates (including aryl–aliphatic, aliphatic, aromatic, and chiral aldoximes) may allow this enzyme class to act as a “bridge builder” in the future between the fine and bulk chemical industries. In particular, for the latter industrial segment, many enzyme classes are still struggling today due to their (relatively) high cost and limited productivity, which makes them, although highly valuable, for example, in the pharmaceutical industry,[^20^, ^21^, ^22^] often not profitable for bulk chemistry applications. The key advances in Oxd catalysis of recent years, with respect to synthetic applications, are summarized in the following.

## Discovery of Oxds

**Figure 1:**
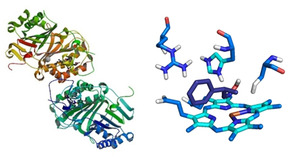
X‐ray structure (left) and the active site (right) of OxdRE, consisting of heme b, including Fe^II^ and serine, histidine, and arginine residues, which represent the catalytic triad.^[37]^

Aldoximes were found to be intermediates in the biosynthesis of certain biologically active compounds,[^23^, ^24^, ^25^, ^26^, ^27^, ^28^] but it took a long time before aldoxime‐degrading enzymes were isolated, purified, and characterized. The first Oxd, namely, OxdB, from *Bacillus* sp. OxB‐1, was discovered in 1998 by screening microorganisms from soil by using (*Z*)‐phenylacetaldoxime ((*Z*)‐PAOx) as a substrate.^[24]^ Such enzymes are known to be involved in nitrile‐degrading microorganisms.[^23^, ^24^, ^25^, ^26^, ^27^] Other enzymes found in this pathway are nitrilases and nitrile hydratases. In addition, plant and millipede P450 monooxygenases also showed Oxd‐like activities.[^28^, ^29^, ^30^] Since the discovery of the first Oxd, five further enzymes of this enzyme class have been found and characterized.[^31^, ^32^, ^33^, ^34^, ^35^, ^36^] These five enzymes were found in *Pseudomonas chlororaphis* B23 (OxdA), *Fusarium graminearum* MAFF305135 (OxdFG), *Rhodococcus erythropolis* (OxdRE), *Rhodococcus globerulus* A‐4 (OxdRG) and *Pseudomonas* sp. K‐9 (OxdK), which are also nitrile degraders. The Oxd enzymes carry a heme b group in their active center, in which a ferrous iron, as a central ion, is crucial for their catalytic activity.[^25^, ^26^, ^31^] An X‐ray structure of OxdRE is shown in Figure 1;^[37]^ the active center, including the heme moiety and the catalytic triad, as key structural features is also schematically shown.

**Scheme 2:**
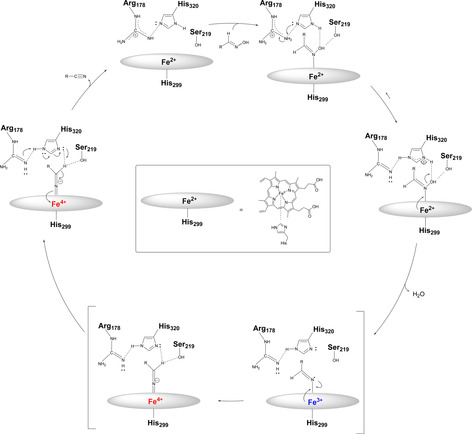
Proposed mechanism of Oxds.

In Scheme 2, the proposed reaction mechanism of Oxd enzymes for the dehydration reaction of aldoximes to nitriles is shown.^[35]^ In case of an oxidation of the heme iron center ion to Fe^3+^, a loss of activity has been observed. A hypothesis for this loss of activity was proposed by Kobayashi et al.,^[35]^ who explained this effect by a redox‐dependent change in the coordination structure of the aldoxime–heme complex. Usually, the aldoxime nitrogen is bound to the iron and, in the case of Fe^3+^ in the active center, the aldoxime binds through the oxime oxygen, which causes a loss of activity. In general, the first step of the mechanism is the coordination of the aldoxime nitrogen to the ferrous iron in the active site of the Oxd and further fixation of the aldoxime substrate by hydrogen bonds between the OH group of the aldoxime and the serine and distal histidine residues. In the next step, the histidine residue is protonated by the arginine residue, which leads to an increase of the electrophilicity of the aldoxime OH functionality. After the elimination of water and electron transfer from the ferrous iron to the aldoxime nitrogen, an Fe^IV^ species is formed and the aldoxime α‐proton is then coordinated to the deprotonated histidine residue and the serine side chain. In the final step, the aldoxime intermediate is deprotonated and an electron transfer to the Fe^IV^ species proceeds, thus releasing the nitrile and recovering the ferrous iron in the ground state. Proton shifts of the histidine and arginine residues complete the catalytic cycle.

**Scheme 3:**
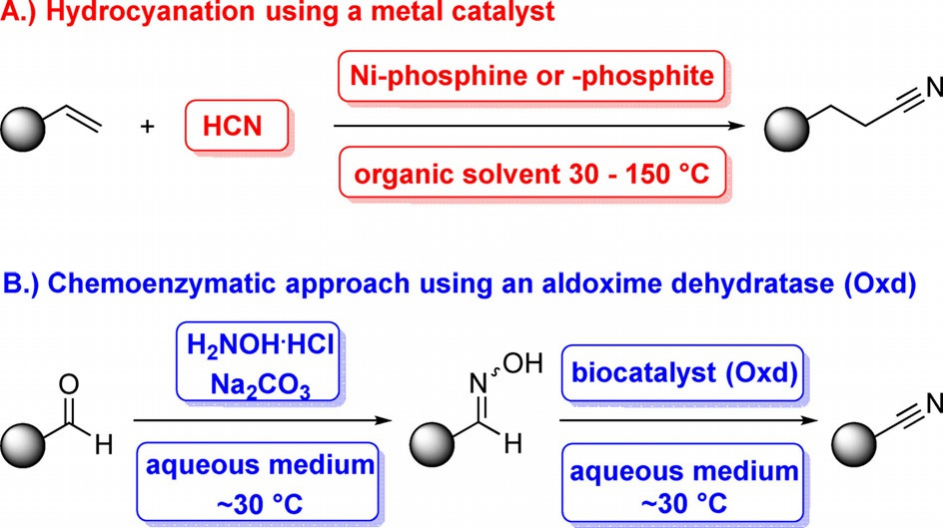
A comparison of hydrocyanation as a common reaction for nitrile synthesis with the chemoenzymatic approach from aldehydes to nitriles by using Oxd as a biocatalyst.

In general, the dehydration of PAOx has been chosen as a standard reaction for the determination of the catalytic activity of Oxds because this substrate was identified as a natural substrate derived from phenylalanine. However, the Oxd enzymes not only accept PAOx as a substrate, but show a broad substrate spectrum. With their catalytic activity and high robustness in synthetic processes, Oxds enable an attractive access to nitriles without the use of toxic cyanide as a reagent. The aldoxime substrates can be easily prepared from the corresponding aldehydes and hydroxylamine as a cheap bulk chemical. In many cases, aldehydes are commercially available or easily accessible from cheap raw materials. For example, aldehydes can be prepared in an elegant fashion through the hydroformylation of alkenes or oxidation of alcohols.[^10^, ^11^] A comparison of the chemoenzymatic synthesis of nitriles from aldehydes by using Oxds for the dehydration of aldoximes and hydrocyanation, as a selected well‐known example of “classical” nitrile synthesis, is shown in Scheme 3.

In the following sections, we describe the capability of Oxd enzymes to act as catalysts in synthetic processes for the cyanide‐free synthesis of nitriles at moderate reaction temperatures in water as a synthetic alternative to form nitriles, thus complementing existing approaches, such as hydrocyanation, ammoxidation, and amide dehydration.

## Enantioselective Synthesis of Chiral Nitriles

**Scheme 4:**
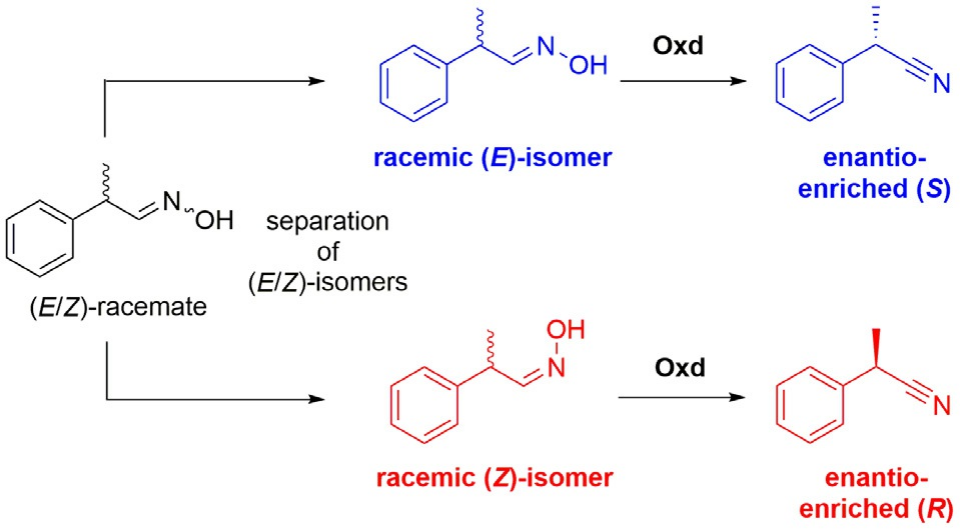
Access to both enantiomers of the same nitrile with the same enzyme, depending on the *E‐* or *Z‐*configuration of the racemic aldoxime.

Oxds have been proven as versatile biocatalysts for the synthesis of chiral nitriles. Initial studies in the early 2000s proved that aldoximes with a chiral center in the α‐position of the oxime moiety were accepted as substrates by many Oxds; however, the stereochemical course of these reactions had not been investigated.[^25^, ^30^, ^31^, ^33^, ^34^, ^36^, ^38^] In this initial work, (*E*/*Z*)‐2‐phenylpropionaldoxime and (*E*/*Z*)‐mandelaldoxime were found to be accepted by up to five different Oxds and showed *K*_m_ values ranging from 1.70 to 11.9 mm, as well as specific activities of 0.57–18.1 U mg^−1^.^[19]^ The stereoselective synthesis of nitriles, starting from racemic aldoximes, was investigated in detail jointly by the groups of Asano and Gröger,^[39]^ who discovered in their preliminary studies that OxdB was able to convert racemic 2‐phenylpropionaldoxime with high enantioselectivity towards (*S*)‐2‐phenylpropionitrile if solely the *E*‐isomer of the aldoxime was used as a racemic substrate.^[18]^ Furthermore, it was found paramount to conduct the biotransformations at 8 °C to suppress the thermal *E* to *Z* isomerization of the aldoxime, since the inversion barrier of the lone pair of nitrogen is rather low. Based on these initial results, we then expanded the substrate scope for this type of chiral nitrile synthesis and the applied biocatalyst toolbox broadly, demonstrating that Oxds did accept a broad range of racemic aldoximes for chiral nitrile synthesis.^[12]^ In particular, substrates with their stereogenic center in the α‐position, as well as a strong steric differentiation of the substituents at the chiral center, showed high enantioselectivity over the reaction course, yielding the corresponding nitriles with up to 99 % enantiomeric excess (*ee*) in these kinetic resolutions. Furthermore, it is noteworthy that Oxds can change their enantiopreference upon changing the substrate from the *E‐* to *Z*‐racemate. This unique stereochemical property has a valuable synthetic consequence, since, with the same enzyme and based on the same racemic aldehyde, both enantiomers are enantioselectively accessible by using either the *E‐* or *Z*‐isomer of the racemic aldoxime as a substrate (Scheme 4).^[12]^

For example, upon separating the *E‐* and *Z*‐isomers of 2‐(3‐bromophenyl)propanal oxime and using them separately in a biotransformation with OxdFG, the (*S*)‐nitrile is obtained in 87 % *ee* at 37 % conversion, if starting from the *E*‐isomer of the aldoxime. However, if the *Z*‐isomer of the aldoxime is used, the (*R*)‐nitrile is obtained with 88 % *ee* at 51 % conversion.^[12]^ This stereochemical phenomenon is, in general, quite unusual in catalysis and, from a synthetic perspective, advantageous because it avoids the necessity to screen for further enzymes with the opposite enantiopreference, if a proper separation of the aldoxime isomers can be conducted.

## Synthesis of Aromatic Nitriles and Utilization of Biorenewable Feedstocks as Raw Materials

**Scheme 5:**
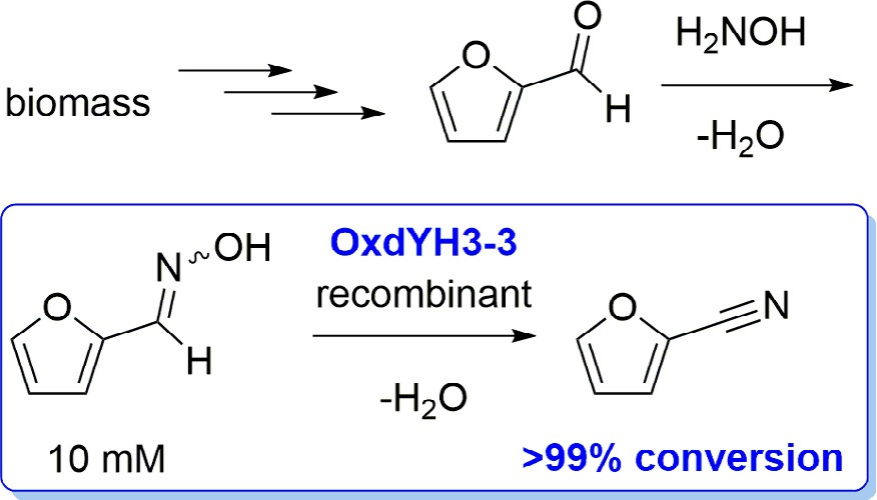
Biocatalytic synthesis of 2‐furonitrile starting from furfural, which can be obtained from biomass.^[41]^

A further class of nitriles important for various industrial segments are aromatic nitriles. Among them, a focus in recent years has been on those that are accessible from biorenewable feedstocks. An example is 2‐furonitrile, which is an intermediate in the field of fine chemicals and pharmaceuticals, as well as a potential sweetener.^[40]^ 2‐Furonitrile can be synthesized, for example, by the ammoxidation of furfural in a gas‐phase process at temperatures of >400 °C.^[40]^ As an alternative approach starting from furfural, which is available from pentoses as a biorenewable raw‐material source, the synthesis of 2‐furonitrile by utilizing Oxd enzymes was reported.[^36^, ^41^, ^42^] The chemoenzymatic synthetic concept and a preparative result are shown in Scheme 5. A task of this study was to gain access to a recombinant form of the Oxd from *Rhodococcus* sp. strain YH3‐3 (OxdYH3‐3). This Oxd was found to convert some aromatic aldoximes and also proved to be suitable to convert furfural oxime.^[36]^ Thus, the gene of OxdYH3‐3 was cloned into expression vectors and expressed in *Escherichia coli*.^[41]^ With this whole‐cell catalyst in hand, 2‐furonitrile was successfully synthesized through this biocatalytic dehydration of 2‐furfuraldoxime (Scheme 5). Because Oxds were usually found to be (mostly) inactive for the conversion of aromatic aldoximes to nitriles,^[19]^ this recombinant OxdYH3‐3 provides a catalyst for practical access towards aromatic nitriles through this enzymatic route.^[41]^

Furthermore, mutants of OxdYH3‐3 were generated by directed evolution, which showed an up to sixfold increased activity for the synthesis of 2‐furonitrile and 3‐cyanopyridine compared with the activity of the wild‐type enzyme.^[42]^

## Synthesis of Aliphatic Nitriles with Utilization as Bulk and Commodity Chemicals

Aliphatic nitriles are a further class of nitriles that are widely used in industry.[^9^, ^43^] Direct applications of such nitriles are particularly known for short‐chain nitriles, such as acetonitrile, whereas a large portion of the longer‐chain aliphatic nitriles serve as intermediates for hydrogenation to the corresponding amines. For example, the resulting fatty amines can be found in many household products, as well as industrial products, with a worldwide demand reported to be 800 000 tons in 2011.^[44]^ Furthermore, here, fats and oils as renewable building blocks can serve as a raw‐material source and, thus, as an alternative to petrochemicals.

Already in early work with Oxds, such enzymes were shown by the group of Asano to catalyze the dehydration of aliphatic aldoximes to give aliphatic nitriles.^[38]^ For example, it was demonstrated that Oxds formed acetonitrile upon starting from acetaldoxime as the smallest substrate for Oxds. When using whole cells with OxdB, a nearly quantitative conversion of 97 % to acetonitrile was observed at a substrate concentration of 0.1 m (≈18 g L^−1^) of acetaldoxime. In the same work, linear aliphatic aldoximes with a chain length between three and six were also found to be converted by different Oxds. At a substrate concentration of 0.3 m (≈35 g L^−1^) hexanal oxime, quantitative conversion was achieved with OxdB as a biocatalyst within 3 h reaction time at 30 °C. However, in addition to linear aliphatic aldoximes, branched ones, such as isobutyraldoxime and isovaleraldoxime, were also found to be converted by Oxds. Based on these initial studies, a detailed process development of aliphatic nitrile synthesis by using OxdB in whole cells as a catalyst was conducted very recently by the group of Gröger, who utilized *n*‐hexanaloxime, *n*‐octanaloxime, and *n*‐decanaloxime as model substrates.^[14]^ Selected results are shown in Table 1, demonstrating the high volumetric productivity of OxdB as a catalyst for aliphatic nitrile synthesis. It is noteworthy that these biotransformations can be conducted at a substrate loading of up to 1.4 kg of aldoxime per liter of aqueous reaction medium, leading to the desired aliphatic nitriles with conversions exceeding 90 % and even reaching quantitative conversion in some cases.

**Table 1:** Preparative biotransformation of aliphatic aldoximes to nitriles by using OxdB in whole cells.^[14]^

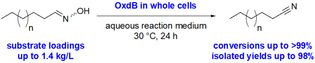				
Entry	*n*	Substrate loading [g L^−1^]	Conversion [%]	Yield [%]
1	1	288	>99	81
2	2	342	>99	84
3	2	428	93	n.d.
4	3	665	>99	98
5	3	1430	93	n.d.

These substrate loadings are among the highest ever reported in biocatalysis, in particular, for conversions of nearly water‐insoluble substrates in aqueous medium. Because the productivity of OxdB for the synthesis of aliphatic nitriles is also very high, this biocatalytic reaction represents a process with promising potential for being transferred to an industrial scale in the future.

Immobilization of Oxds for applications in aqueous medium was also studied, revealing high stability of OxdB whole cells entrapped in calcium alginate beads and coated with tetraethoxysilane.^[45]^

**Scheme 6:**
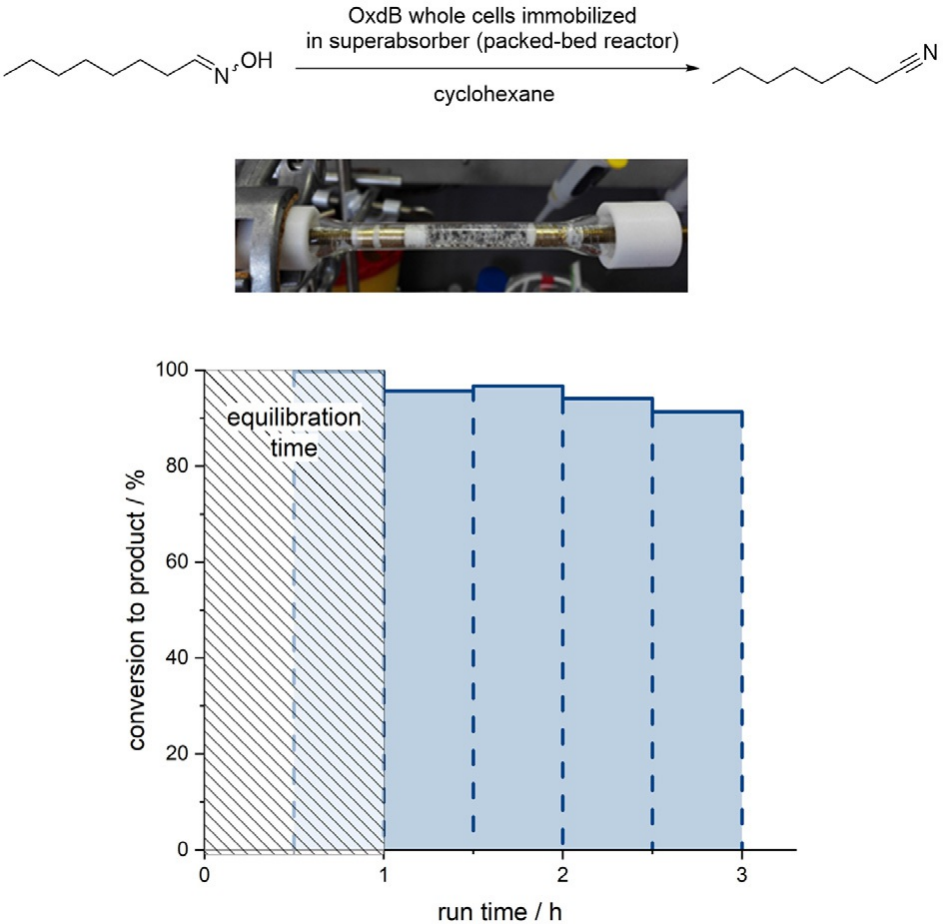
Flow‐based biocatalytic synthesis of *n*‐octanenitrile by means of OxdB whole cells immobilized in superabsorber and used in a packed‐bed reactor.^[15]^ The photo and graphic of this scheme is reproduced from reference [15].

Furthermore, Oxds were found to be active in pure organic solvent if a superabsorber‐based immobilization technique was used (Scheme 6).[^15^, ^46^]

Because many aldoximes, especially long‐chain aliphatic aldoximes, are barely soluble in aqueous reaction medium, this possibility enables the use of this Oxd biocatalyst in organic medium, in which the aldoxime is better soluble. This superabsorber‐immobilized Oxd catalyst turned out to be suitable for application in a continuous process with a packed‐bed reactor, showing sufficient stability and, thus, high remaining activity for a run time of at least 3 h (Scheme 6).^[15]^ Furthermore, this Oxd catalyst, which is entrapped in a superabsorber, can also be applied as a fluid heterogeneous phase in a segmented flow mode.^[46]^

**Scheme 7:**
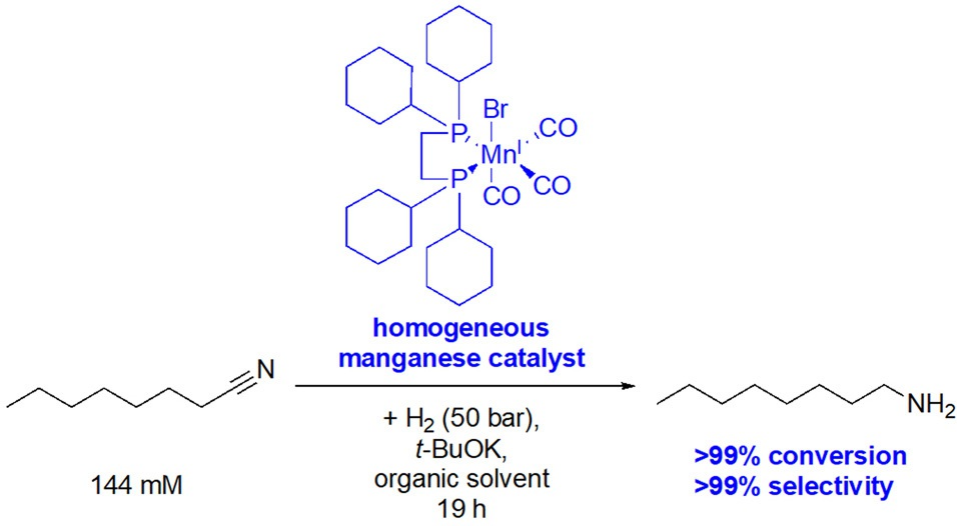
Hydrogenation of *n*‐octanenitrile by using a Mn^I^‐based catalyst, yielding *n*‐octan‐1‐amine with high selectivity and conversion.^[47]^

Because, in industry, most aliphatic nitriles, in particular, fatty nitriles, are utilized as intermediates for the production of the corresponding amines,^[43]^ the development of novel hydrogenation methods for their synthesis is also a field of current interest. Industrial valuable primary aliphatic amines can be synthesized starting from these aliphatic nitriles by hydrogenation. However, the hydrogenation of nitriles in a very selective manner is still a challenge, especially if utilizing heterogeneous catalysts.^[47]^ The group of Kirchner,^[48]^ and based on their work recently also our group,^[47]^ investigated homogeneous manganese catalysts for such transformations with high selectivities. For example, *n*‐octanenitrile can be selectively hydrogenated to *n*‐octan‐1‐amine with >99 % conversion and >99 % selectivity by using such a manganese catalyst (Scheme 7).^[47]^

In addition, researchers from BASF reported the application of Oxds for the synthesis of citronellyl nitrile, which is used as a fragrance compound.^[49]^ In these reactions, running under neat conditions directly in citronellal oxime, the Oxds were also used as whole‐cell catalysts, leading to quantitative conversion after 90 h at 30 °C. This example underlines the high industrial potential of Oxds and it shows the opportunity to use Oxds in a pure organic system under neat conditions, thus leading to high space–time yields.

**Scheme 8:**
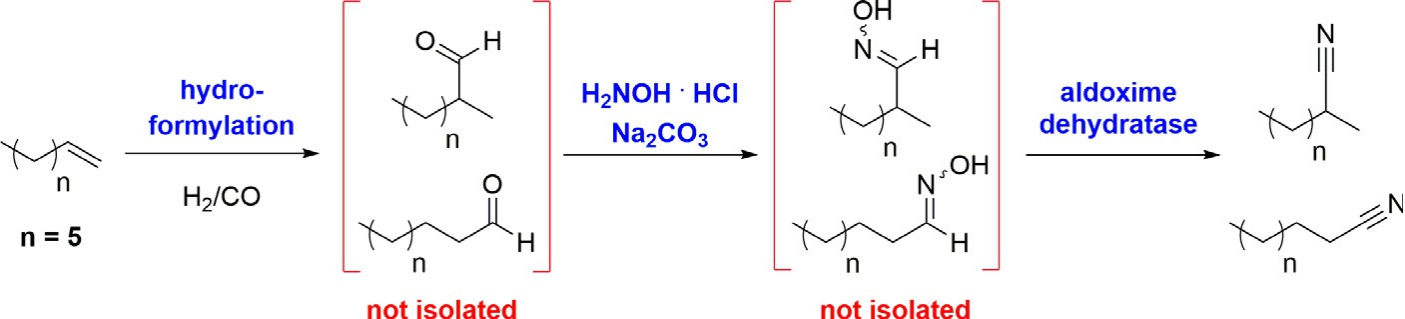
Chemoenzymatic cascade from 1‐octene towards nonanenitrile and 2‐methyl‐octanenitrile without intermediate isolation and purification.^[16]^

In addition to process development of individual reaction steps, process integration of various reaction steps towards cascades without the need for intermediate purification represents a versatile concept for industrial production. Addressing the combination of enzymatic and classic chemical and chemocatalytic reactions,^[50]^ a chemoenzymatic cascade with Oxds for aliphatic nonanenitrile synthesis, starting from 1‐octene as a readily available raw material, was developed.^[16]^ This chemoenzymatic cascade combines the hydroformylation of 1‐octene to *n*‐/iso‐C_9_‐aldehydes with subsequent condensation of these aldehydes with hydroxylamine under the formation of aldoximes, followed by the biocatalytic dehydration of the aldoximes to the nitriles (Scheme 8).^[16]^ The initial hydroformylation step was conducted in a biphasic system consisting of water and 1‐octene by using a rhodium complex with the commercial triphenylphosphine‐3,3′,3′′‐trisulfonic acid trisodium salt (TPPTS) ligand as a catalyst. The catalyst is water soluble, thus enabling a simple separation of the organic product–substrate mixture from the catalyst. After this phase separation, the organic phase mainly consists of *n*‐nonanal and 2‐methyloctanal as products, resulting from the hydroformylation of 1‐octene and an isomer of 1‐octene. This mixture was then treated with hydroxylamine in aqueous reaction medium, followed by heating overnight to remove residual traces of hydroxylamine, which causes deactivation of the Oxd. Subsequently, the aldoxime mixture was converted by an Oxd using an aqueous reaction medium. Using this chemoenzymatic cascade reaction, an overall conversion of 67 % and a yield of the desired *n*‐/iso‐C_9_‐nitriles of 41 % was obtained, thus demonstrating that after optimization the Oxd‐based biotransformation was also compatible with the chemical reaction steps for the synthesis of the aldoxime substrates.

## Synthesis of Nitrile‐Substituted Fatty Acids

**Scheme 9:**
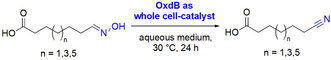
Chemoenzymatic synthesis of terminal aliphatic nitriles bearing a carboxylic acid moiety by using OxdB as a biocatalyst.^[51]^

Furthermore, Oxd enzymes have also been successfully applied in the synthesis of terminal aliphatic nitriles bearing a carboxylic acid moiety as a further functional group (Scheme 9).^[51]^ Such bifunctional molecules are of interest for polymer production because the nitrile group can be subsequently converted into an amino moiety by means of hydrogenation, thus furnishing ω‐amino‐substituted fatty acids as monomers for polyamides.

Access to the aldoxime substrates was realized by means of the hydroformylation of terminal aliphatic alkenes and subsequent treatment with hydroxylamine. The resulting aldoximes were then converted into the desired cyano‐substituted fatty acids with quantitative conversion at a substrate concentration of 10 mm, whereas the conversions decreased with increasing substrate concentration, leading to a conversion of less than 50 % at substrate concentrations of 100 mm and above. This approach is also suitable for the synthesis of nitrile‐substituted aliphatic carboxylic acids (and thus, ω‐amino acid polymer precursors) starting from unsaturated fatty acids as biorenewable feedstocks. For example, cross‐metathesis with oleic acid and ethylene furnishes dec‐9‐enoic acid, which then is transformed, by means of hydroformylation and subsequent oxime formation, into the C_11_‐aldoxime as a substrate for enzymatic dehydration. This biotransformation then gives a C_11_‐carboxylic acid bearing a nitrile moiety as a substituent.

## Synthesis of Aliphatic Dinitriles

**Scheme 10:**
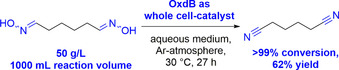
Adiponitrile synthesis in water at 30 °C on the liter scale by using OxdB as a biocatalyst.^[13]^

Another potential application area for the industrial use of Oxds in the field of commodity chemicals is the biocatalytic synthesis of aliphatic dinitriles.^[13]^ Among them, adiponitrile is the one with the highest production volume, being in the range of about one million tons annually.^[9]^ Adiponitrile is utilized mainly for the manufacture of 1,6‐hexanediamine as a monomer for polymerization with adipic acid to give nylon‐6,6, which is produced on the multimillion‐ton scale per year. As an alternative to current existing methods, such as the hydrocyanation of butadiene, as the main applied process in terms of production volume, recently the capability of Oxd enzymes for transforming the bis‐aldoxime of adipaldehyde into adiponitrile was successfully demonstrated (Scheme 10).

This cyanide‐free approach towards adiponitrile is conducted in water as a solvent and at a low reaction temperature (30 °C). The process has been already demonstrated on a 1 L reaction scale with 50 g L^−1^ substrate loading, leading to full conversion within 27 h in the presence of a recombinant whole‐cell catalyst bearing OxdB as a biocatalyst.

Furthermore, other linear dinitriles (C_4_–C_10_) were shown to be converted by Oxds under similar reaction conditions. The bis‐aldoxime substrates can be synthesized starting from the corresponding bis‐aldehydes or their acetal‐protected derivatives. These examples underline that Oxds also accept bis‐aldoximes as substrates very well, as exemplified for linear aliphatic representatives of this compound class.^[13]^ Recently, not only the oxidation of monoalcohols to aldehydes, but also the oxidation of diols to dialdehydes, was successfully performed by means of a (2,2,6,6‐tetramethylpiperidin‐1‐yl)oxyl‐catalyzed oxidation in nitriles as a solvent.^[11]^

## Summary and Outlook

Although their synthetic potential has only recently been studied more intensively, Oxds have already proven to be highly capable for the synthesis of chiral nitriles, aromatic nitriles, aliphatic nitriles, and aliphatic dinitriles. In case of chiral nitriles, such enzymes are able to yield both enantiomers of a nitrile, despite using the same enzyme, due to the dependency of the enantiopreference on the *E*‐ and *Z*‐configuration of the utilized racemic aldoxime substrate, thus enabling the opportunity to gain access to both enantiomers of a chiral nitrile building block with the same enzyme. Furthermore, the potential of Oxd enzymes for the synthesis of bulk chemicals has been demonstrated by the liter‐scale synthesis of the polyamide intermediate adiponitrile, with up to 50 g L^−1^ substrate loading. In addition, Oxds have been implemented in a chemoenzymatic reaction cascade to obtain fatty nitriles, starting from alkenes in combination with hydroformylation. Fatty nitriles, as a further nitrile product class of industrial interest, can be prepared by Oxds in a very productive manner at substrate loadings of up to 1.4 kg L^−1^ of reaction medium. For the future, we expect that the utilization of Oxds in the cyanide‐free synthesis of nitriles under mild reaction conditions will gain further increasing interest for preparing nitrile products from various segments of the chemical industry, such as commodity chemicals, bulk and fine chemicals, and pharmaceuticals.

## Conflict of interest

The authors declare no conflict of interest.
